# Enhanced Sensor Placement Optimization and Defect Detection in Structural Health Monitoring Using Hybrid PI-DEIM Approach

**DOI:** 10.3390/s25010091

**Published:** 2024-12-27

**Authors:** Minyoung Yun, Mikhael Tannous, Chady Ghnatios, Eivind Fonn, Trond Kvamsdal, Francisco Chinesta

**Affiliations:** 1 PIMM Research Laboratory, UMR 8006 CNRS-ENSAM-CNAM, Arts et Metiers Institute of Technology, 151 Boulevard de l’Hôpital, 75013 Paris, France; minyoung.yun@ensam.eu (M.Y.); mikhael.tannous@ensam.eu (M.T.); francisco.chinesta@ensam.eu (F.C.); 2 Mechanical Engineering Department, University of North Florida, 1 UNF Drive, Jacksonville, FL 32224, USA; 3 Department of Mathematics and Cybernetics, SINTEF Digital, Kloebuveien 153, 7465 Trondheim, Norway; eivind.fonn@sintef.no (E.F.); trond.kvamsdal@ntnu.no (T.K.); 4 Department of Mathematical Sciences, Norwegian University of Science and Technology, Alfred Getz’ vei 1, 7491 Trondheim, Norway; 5 CNRS@CREATE Ltd., 1 Create Way, #08-01 CREATE Tower, Singapore 138602, Singapore

**Keywords:** random permutation features importance method, optimal sensor placement, discrete empirical interpolation method, machine learning

## Abstract

This work introduces a novel methodology for identifying critical sensor locations and detecting defects in structural components. Initially, a hybrid method is proposed to determine optimal sensor placements by integrating results from both the discrete empirical interpolation method (DEIM) and the random permutation features importance technique (PI). Subsequently, the identified sensors are utilized in a novel defect detection approach, leveraging a semi-intrusive reduced order modeling and genetic search algorithm for fast and reliable defect detection. The proposed algorithm has successfully located defects with low error, especially when using hybrid sensors, which combine the most critical sensors identified through both PI and DEIM. This hybrid method identifies defects with the lowest errors compared to using either the PI or DEIM methods alone.

## 1. Introduction

Damage, often characterized as adverse changes within structural systems, poses significant concerns for infrastructure integrity across various fields, ranging from aerospace to civil and mechanical engineering. The effective monitoring and timely identification of defects are pivotal components of structural health monitoring (SHM) practices, essential for ensuring the safety and longevity of structures [1,2]. To achieve the earliest possible detection of structural defects, continuous monitoring is essential, facilitated by sensors. However, the deployment of sensors is constrained due to factors such as installation and maintenance costs, necessitating the optimization of sensor placement [3]. Recent advances have demonstrated the effectiveness of piezoelectric sensors and their configurations for structural damage detection and localization, providing valuable insights into optimal sensor placement [4,5,6]. With limited resources available for sensor installation and maintenance, identifying optimal sensor locations becomes vitally important [7].

Previous research has extensively explored sensor placement strategies for structural health monitoring [8,9,10,11,12,13]. An illustrative example of sensor placement methodology is the discrete empirical interpolation method (DEIM), designed to efficiently approximate nonlinear and dynamic systems [14,15]. DEIM leverages the availability of the proper orthogonal decomposition (POD) modes, in a vector form, of the system at hand. Each mode identifies a potential sensor location, typically starting with the most influential mode, such as the one with the highest energy. While DEIM offers simplicity and physical underpinnings, there remains room for enhancement. Specifically, given that only a limited number of nodes are typically identified, often just a few from the most influential POD modes, we propose leveraging not only the information from POD modes but also the associated coefficients generated from the POD calculation.

The permutation features importance (PI) method [16], DEIM, effective independence (EfI) [17], and Shannon information-based [18] approaches all serve to optimize sensor placement, but they differ in their core principles and applications. The PI method focuses on refining sensor locations by prioritizing the output sensitivity to input parameters, while DEIM leverages reduced-order models, selecting sensor locations based on a reduced basis constructed using the proper orthogonal decomposition (POD) modes for computational efficiency. EfI aims to maximize the independence of measured data, ensuring that the sensors provide uncorrelated and diverse information. Shannon information-based approaches use information theory to select sensors that maximize the entropy or mutual information, optimizing for data informativeness or the quantity of relevant information that is conveyed. Compared to EfI and Shannon information-based approaches, PI and DEIM are computationally efficient for large-scale systems due to their reliance on reduced-order modeling, whereas EfI and Shannon approaches emphasize data quality but may involve higher computational costs. Other methods, like convex optimization-based [19] and greedy algorithms [20], have also been explored, often trading off between computational demands and optimality. This variety of methodologies highlights the trade-offs between accuracy, computational efficiency, and ease of implementation.

The PI method demonstrated by R. Semaan [21] exemplifies the use of the expansion coefficients from POD through the permutation features importance (PI) method with random forest (RF). In this approach, the most influential nodes identified via PI are selected as sensor locations. Building upon this foundation, our study introduces a hybrid methodology that combines the outputs of both the PI and DEIM methods. By integrating information from both techniques, we aim to identify optimal sensor placements by combining the comprehensive insights of the PI with the structural approach available in DEIM, positioning the hybrid PI-DEIM methodology as an efficient and scalable option for industrial applications. The efficiency of the selected sensors from the novel hybrid method is tested and confirmed in this study, using a defect detection method. As with many prior studies, it is essential to provide convincing evidence that these sensors are strategically positioned for effective defect detection. To address this need, our study employs a novel defect detection method equipped with lightly intrusive reduced order modeling (ROM) [22] and a genetic search algorithm (GA). This methodology enables structural calculations to be executed within seconds, significantly reducing the computation time compared to conventional defect detection approaches using direct finite element calculations. The rapid computational process allows for integration with global search algorithms such as GA, facilitating efficient defect identification.

To sum up, this study introduces a novel hybrid sensor placement method, which improves upon conventional approaches such as the DEIM or PI methods used individually. Additionally, the study conducts defect detection using a novel search method that combines a lightly intrusive ROM with GA. By employing this defect detection method, the study demonstrates the efficiency and effectiveness of the proposed sensor placement methodology. This paper is structured into four main sections: Introduction, Method, Results, and Discussion, followed by Conclusions.

## 2. Method

**Figure 1:**
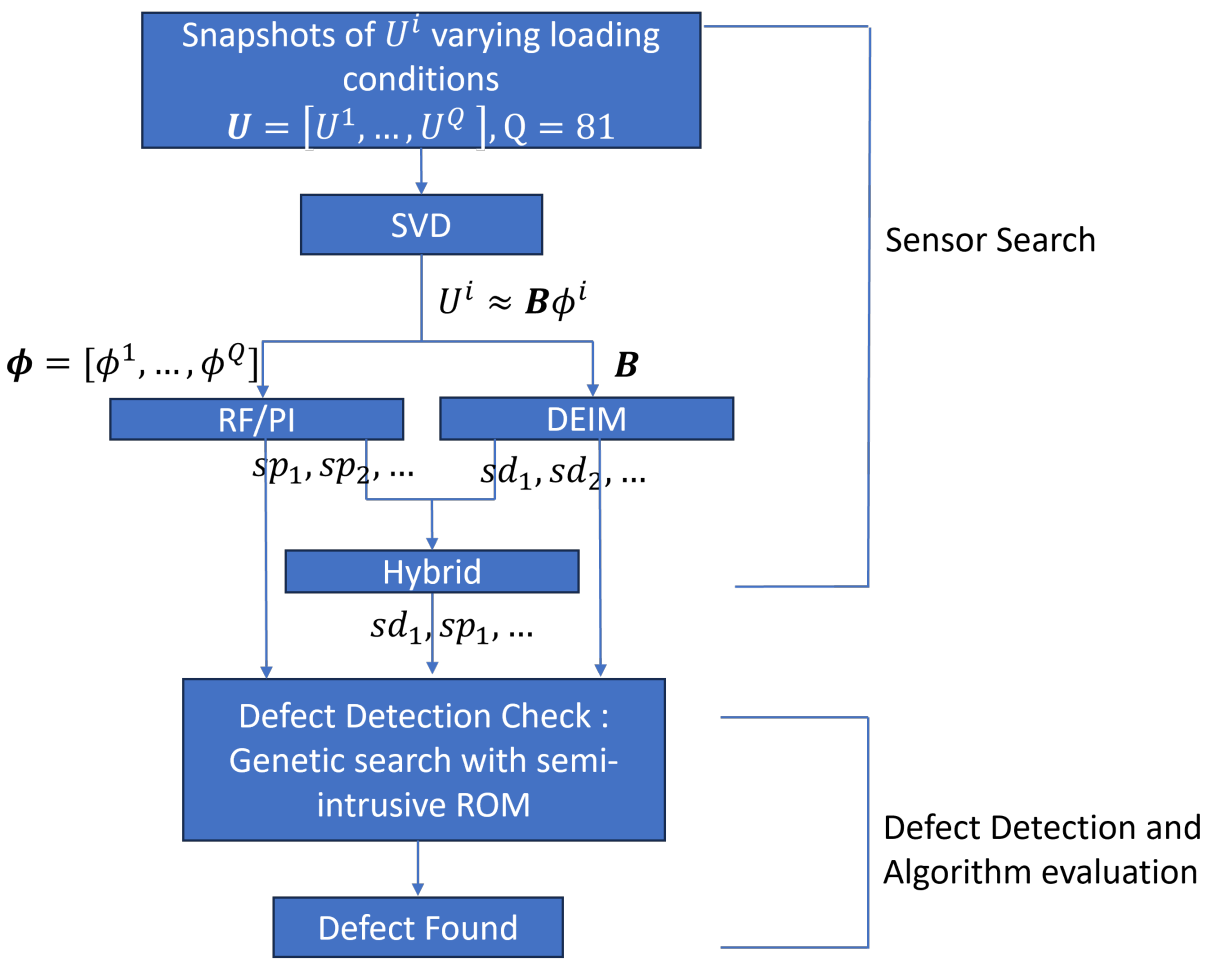
Flowchart of the developed hybrid PI-DEIM methodology in this study. Here, RF/PI is the random forest/permutation features importance method, and DEIM is the discrete empirical interpolation method.

The methodology used in this work comprises two primary components: sensor search and defect detection, as illustrated in Figure 1. In the initial phase, sensor locations are identified using a hybrid model combining the results from the PI and DEIM methods. Subsequently, defect detection is performed using a lightly intrusive ROM coupled with a GA, utilizing the selected sensors.

The sensor search involves acquiring displacement snapshots, $U^{i}$, under varying loading conditions and for a given combination of parameters, $\mu^{i}$. Once the snapshots $U^{i}$ are available, we proceed by decomposing them into POD mode vectors via singular value decomposition (SVD):

(1) $$\left[V,\xi ,S\right]=svd\left(U\right)$$

With V being the left singular vectors, W the right ones, and ξ the matrix of singular values. The diagonal elements of ξ are denoted as $\xi_{i}$. The left singular vectors matrix V is truncated using the singular values at a rank *n*, such as the following:

(2) $$\frac{\sum_{i=1}^{n}\xi_{i}}{\sum_{i=1}^{Q}\xi_{i}}>0.99$$

with *Q* being the total number of vectors in V. The truncated basis is denoted as B, which will be leveraged in both the PI method and the POD-DEIM one to determine the critical sensor locations for defect detection. The sensor locations identified via the PI method are denoted as $sp_{i}$, and those identified via the DEIM method are denoted as $sd_{i}$, as mentioned in Figure 1. The hybrid method selects, later on, the most significant sensors, for example, $sp_{1}$ and $sd_{1}$, from both approaches by studying the POD and PI scores. In the defect detection step, the genetic algorithm is applied, coupled with the lightly intrusive ROM, to reconstruct the solution. The genetic algorithm accesses the sensors’ measurements, identified via PI, DEIM, and the hybrid method, to reconstruct the complete solution through identifying the exact problem properties. The error in the detected parameters from each method is used for comparison to assess the proposed approaches.

The following sections provide a detailed explanation of these two parts.

### 2.1. Sensor Search

#### 2.1.1. Constructing the Data Set and the Reduced Basis

POD is employed to represent the original data in a low-dimensional space. The SVD of snapshots matrix $U=\left[U^{1},\ldots ,U^{Q}\right]\in R^{N\times Q}$ yields orthogonal basis vectors and a diagonal matrix of singular values [23,24].

(3) $$U=V\xi S^{T}$$

$\xi_{i}$ are the singular values of U, which is also the weight (or importance) of each one of the POD modes. Using the truncated basis vectors B, *U* can be approximated as follows:

(4) U≈Bϕ,

with $B\in R^{N\times n}$. ϕ is the vector of the reduced coordinates of the system. Therefore, the original data can be truncated and compressed with the optimal number of *n*, n≪N.

**Figure 2:**
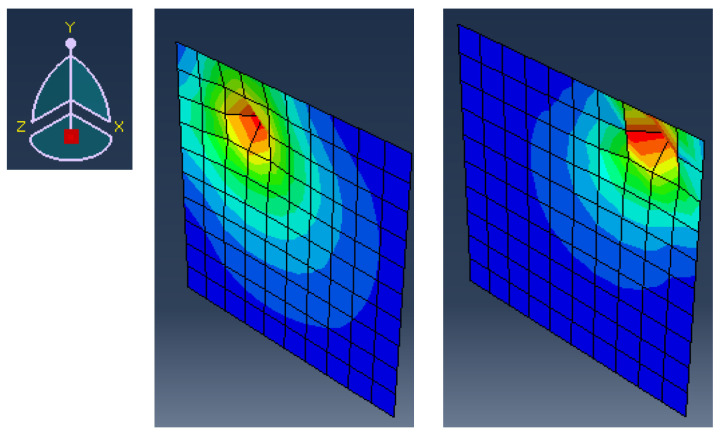
Selected displacement examples, $U_{i}$, for two different set of parameters, of a square plate subject to varying concentrated force locations $\left(X^{i},Y^{i}\right)$. Displacement values are scaled for an enhanced visual representation.

In this study, the original data that will be used to create the reduced basis consist of snapshots of displacements under a varying concentrated force location. For instance, we build a database such as the following:

(5) $$U^{i}=f\left(X^{i},Y^{i}\right),$$

where the displacement $U^{i}$ is a function of the position of the applied force in the space $\left(X^{i},Y^{i}\right)$. The solution is computed for all possible combinations of $\left(X^{i},Y^{i}\right)$. Numerical simulations are conducted using commercial finite element software, modeling a horizontal plate supported on its edges, subjected to vertical loading. The boundary conditions constrain all the displacements along the edges, excluding them from consideration as concentrated force locations. Selected examples of these displacement snapshots are illustrated in Figure 2, and they represent the plate’s response to concentrated force at different selected positions, $\left(X^{i},Y^{i}\right)$. The displacements of interest are mainly those perpendicular to the plate (along the z-axis). The local effect of concentrated force can be seen in Figure 2, although it is scaled to enhance the visual representation. The study cases treated in this paper are limited to quasi-static loading.

#### 2.1.2. Permutation Features Importance Method (PI)

PI measures the change in prediction accuracy resulting from random permutations of the input variables after a random forest (RF) is trained. Conceptually, PI resembles the derivative of outputs with respect to inputs in RF [25,26,27].

In this study, the RF model was trained to predict the reduced coordinates ϕ in order to approximate the displacement vectors *U*, such as the following:

(6) $$\phi^{i}=f_{RF}\left(U^{i}\right),$$

with $f_{RF}$ being the trained RF surrogate model. Once the RF model is trained, its prediction accuracy is assessed by introducing random variations in $U^{i}$, at a given position $\left(x_{j},y_{j}\right)$, for $j\in 1,\ldots ,\bar{N}$. $\bar{N}$ is the total number of nodes excluding the Dirichlet boundary conditions. The importance score, $W_{j}$, is attributed for each node, *j*, using this technique by observing the changes in the prediction accuracy when the nodal value *j* is randomly shuffled. For instance,

(7) $$W_{j}=\left|E_{j}-E\right|,$$

with E the prediction error of $f_{RF}$ when using the correct inputs, and $E_{j}$ is the error generated via the same trained $f_{RF}$ surrogate when the input values of node *j* are replaced with wrong, randomly shuffled values. The error E is defined using the following:

(8) $$E=∥V\phi -V\;f_{RF}\left(U\right)∥,$$

where $f_{RF}\left(U\right)$ refers to the matrix that contains the application of model (6) for each displacement vector, *U*, and $\phi =V^{T}U$ is the matrix of all the reduced coordinates of the training data set.

This PI process ranks the most influential nodes, $sp_{j}$, for the RF model’s predictions, the ones having the highest importance, $W_{j}$. For instance,

(9) $$sp_{1}=j\mathrm{such}\mathrm{as}W_{j}=max\left(W\right)$$

with W being the vector of all $W_{j}$ values. The process repeats for the next most relevant node by excluding the components already identified.

PI computes the importance of all candidate sensors, which are all the nodes *j* in the mesh of size $\bar{N}$. The use of the PI method allows a comprehensive exploration of potential nodal points for optimal sensor placement. This global perspective differs from DEIM, where sensors are identified from individual POD modes, and the relative importance among potential candidates is not clearly provided.

#### 2.1.3. Discrete Empirical Interpolation Method (DEIM)

The empirical interpolation method was devised to efficiently obtain an approximation of the non-linear terms in dynamic structural analysis by interpolating using a reduced number of interpolation points [28,29]. The interpolation points used in this model-order reduction technique are found in the reduced basis vectors identified using the POD method and applied to the system of interest. For DEIM, the snapshots of displacement vectors $U^{i}$ are first decomposed by SVD, and the results are truncated to produce reduced basis vectors, B. The sensor locations are, later on, found leveraging B to construct an index matrix, P. The first selected sensor point, $sd_{1}$, is selected at the node having the highest value of the most dominant vector in B. For instance,

(10) $$sd_{1}=j\mathrm{such}\mathrm{as}B_{j1}=max\left(B_{\kappa 1}\right);\kappa \in \left[1,N\right]$$

The first column of matrix P has only one non-zero element, at location $sd_{1}$, which corresponds to the first sensor location, while all the other elements in that column are set to zero. The subsequent sensor points ($sd_{2},\ldots ,sd_{Q}$) are selected by computing the residual; for example, for the ${\left(q+1\right)}^{th}$ sensor, $r_{q+1}$ is computed using the following:

(11) $$\begin{array}{c} P^{T}B^{q}\gamma =P^{T}B^{q+1}\\ r_{q+1}=B^{q+1}-B^{q}\gamma \end{array}$$

In Equation (11), $B^{q}$ are the first *q* vectors of the matrix B, $B^{q+1}$ is the vector number q+1, and γ is a weight matrix to be computed using the first equation.

The following sensor location, q+1, is assigned to the node where the max value of the residual $r_{q+1}$ is found:

(12) $$sd_{q+1}=max|r_{q+1}|$$

As each interpolation point, representing a sensor location, is determined from each $B^{q}$, it becomes necessary to ascertain the number of required POD modes. This can be determined by examining the singular values of U.

#### 2.1.4. Hybrid Method

Once sensors are identified through both the PI and DEIM methods, the most critical sensors are chosen from each method. The degree of importance is determined using the PI score for PI sensors and the singular values of POD for DEIM sensors.

To select the most relevant sensors in the hybrid model, the sensor contribution can be first normalized independently in each method using the following:

(13) $$w_{j}^{PI}=\frac{W_{j}}{\sum_{k=1}^{\bar{N}}W_{k}}$$

and

(14) $$w_{j}^{DEIM}=\frac{∥r_{j}∥}{\sum_{k=1}^{\bar{N}}∥r_{k}∥}$$

with $\bar{N}$ being the total number of possible sensors, $w^{PI}$ and $w^{DEIM}$ are the normalized sensor weights for the PI and DEIM methods, respectively.

### 2.2. Defect Detection

Defect detection is performed using a genetic algorithm in conjunction with a lightly intrusive reduced order model (ROM), which computes displacements under static loading conditions in real time for each parameter combination [22].

The computational efficiency of the genetic algorithm is heavily influenced by the cost of the displacement calculations required to evaluate the sensor positions during the iterative search. For complex geometries, this process becomes increasingly computationally demanding. By employing the lightly intrusive methodology, which enables a real-time computation of displacements, the search process is significantly accelerated, making it viable for complex industrial applications. For the simple geometry considered in this study, the lightly intrusive method achieves a speedup of approximately sixty times compared to a conventional finite element solver.

#### 2.2.1. Lightly Intrusive ROM

The lightly intrusive ROM is obtained through the application of the POD method, followed by another projection of the system matrix [22]. This involves using the Galerkin projection, which projects the full-order solution and its reshaped stiffness matrix onto a sub-space obtained from its snapshot version. The ROM, once established, is capable of approximating the full-order solution within a small error range, significantly reducing the computation time [30]. For this defect detection study, a linear static problem is chosen as an application, for the sake of simplicity and without loss of generality. To create the snapshots, the displacement fields, U, are generated with multiple combinations of the defect parameters.

The general discrete form of the method is presented as follows. First, the partial differential equation is discretized into the final system of equations, written as $K^{i}U^{i}=F^{i}$, for a given combination of the problem’s parameters, *i*, denoted as $\mu^{i}$. With the stiffness matrix $K^{i}$, the nodal displacement $U^{i}$ and force vectors $F^{i}$ are of size N×N, N×1 and N×1, respectively. Nodal displacements and forces can be translated into a subspace of dimension, n≪N. If we consider $U^{i}$ with varying defect parameters, the truncated POD application on the collected full-order solutions $\left(U^{1},\ldots ,U^{Q}\right)$ yields the most relevant orthogonal singular vectors with the highest singular values. The singular vectors in the form of matrix B lead to the full-order snapshots in a reduced map, $U^{i}\approx B\phi^{i}$. The reduced number of the obtained modes, *n*, defines the dimension of the problem, such as the nodal displacements and forces, n≪N.

The introduction of reduced bases leads to the following equation:

(15) $$K^{i}B\phi^{i}=F^{i}.$$

By multiplying both sides with $B^{T}$, the matrix form of $B^{T}K^{i}B$ appears on the left side, leading to the following:

(16) $$B^{T}K^{i}B\phi^{i}=B^{T}F^{i}.$$

Subsequently, the reduced stiffness matrix term, $B^{T}K^{i}B$, is named as $K^{i}$. This can be rewritten as

(17) $$K^{i}\phi^{i}=f^{i}.$$

To construct parametric reduced elasto-statistics, the reduced stiffness matrices $K^{i}$ are reshaped in a vector form to form snapshots for another POD reduction round:

(18) $$K^{i}\to \chi^{i}=Vect\left(K^{i}\right).$$

The stiffness vectors are concatenated into a matrix, χ$=[\chi^{1},\ldots ,\chi^{Q}]$, whose truncated SVD decomposition yields the most relevant orthogonal singular vectors basis, $A=[A^{1},\ldots ,A^{m}]$. With the orthogonal basis, the reduced form of $\chi^{i}$ can be expressed as follows: $\chi^{i}\approx A\psi^{i}$.

A supervised machine learning method, like RF, is trained using defect parameters as input and the corresponding coefficient vector $\psi =[\psi_{1},\ldots ,\psi_{m}]$ as output.

In this work, we build the defect database, such as the following:

(19) $$U^{i}=h\left(E^{i},c^{i},\alpha^{i},\beta^{i}\right),$$

with $E^{i}$ being the modulus of elasticity inside a square defect, $c^{i}$ is the side length of the defective square, $\alpha^{i}$ and $\beta^{i}$ are the *x* and *y* coordinates of the defect’s center. A data set of multiple simulations is built, and later on, a random forest algorithm is trained to predict ψ using the following:

(20) $$\psi =h_{RF}\left(E,c,\alpha ,\beta \right).$$

In this work, we note the defect parameters vector $\mu^{i}=\left[E^{i},c^{i},\alpha^{i},\beta^{i}\right]$. With ψ readily available using model (20), the stiffness matrix is evaluated in almost real time to obtain the displacement fields for any combination of parameters, μ.

On the other hand, defect detection relies on a lightly intrusive approach to construct the displacement solution, inheriting some limitations of the approach itself. As demonstrated in [22], this method has proven to be accurate for a variety of linear and geometrically nonlinear problems, including those involving complex geometries. However, it has yet to establish its efficacy for history-dependent nonlinear problems, such as material plasticity. A modified version of the method is anticipated to address these nonlinear aspects, which could significantly extend its applicability.

If such advancements are achieved, the defect detection approach outlined in this paper stands to benefit, enabling it to tackle more complex and highly nonlinear challenges. For the current scope, the method is effective for detecting defects that can be modeled using linear problems, such as variations in the modulus of elasticity, thickness, or Poisson’s ratio. This capability extends to geometrically complex structures subjected to intricate loading conditions or geometric nonlinearity, provided that the normal operating conditions of the structure do not involve severe nonlinearity, particularly history-dependent material behaviors.

#### 2.2.2. Genetic Search Algorithm

GA, rooted in the principles of natural selection, stands as a widely popular tool for global search optimization [31,32]. The optimization process involves three main operations: mutation, selection, and crossover, which iteratively refine parameters within the solution space.

In this study, GA was used to identify defect parameters μ, including the location, size, and material property. To conduct the global search, a defined range for each parameter, normalized from 0 to 1, is provided, $\mu_{nr}\in {\left[0,1\right]}^{4}$. $\mu_{nr}$ is the normalized defect parameter. A heuristic approach to generating random initial guesses was developed, and parameter pools were built to minimize the cost function. The selected cost function, J, uses the $L_{1}$ norm of the difference between two vectors, specifically the measured sensor displacements ${\hat{U}}_{real}$ and the predicted displacement ${\hat{U}}_{pred}$ at the sensor location:

(21) $$J\left({\hat{U}}_{real},{\hat{U}}_{pred}\right)=\left|\right|{\hat{U}}_{real}-{\hat{U}}_{pred}{\left|\right|}_{1}$$

The genetic algorithm was implemented using Python’s differential evolution function. Various GA parameters, such as population size and mutation range, were tested to optimize the search. During the optimization process, each one of the three parameters was normalized using a min–max scaler, the population size was limited to 10, and the number of generations or interactions was limited to 100. Dithering was used for the mutation constants, and the cross-match threshold was set to 0.7.

#### 2.2.3. Sensor Confirmation

A sensor confirmation step was devised to assess the efficiency of the sensors identified via PI, DEIM, and the hybrid method. A total of 45 cases were designed by varying $\mu^{i}$ and the location, size, and Young’s modulus of defects using a design of experiments approach. The efficiency of the sensors from each method across all 45 cases was tested and calculated. Efficiency was defined using the defect identification error, *e*, which is the mean of the $L_{1}$ norm of the difference between actual values, $\mu_{real}^{i}$, and predicted values, $\mu_{pred}^{i}$:

(22) $$e=\frac{1}{45}\sum_{i=1}^{45}{∥\mu_{real}^{i}-\mu_{pred}^{i}∥}_{1}.$$

A smaller error, *e*, indicates a higher efficiency for the method with the sensors.

## 3. Results and Discussion

**Figure 3:**
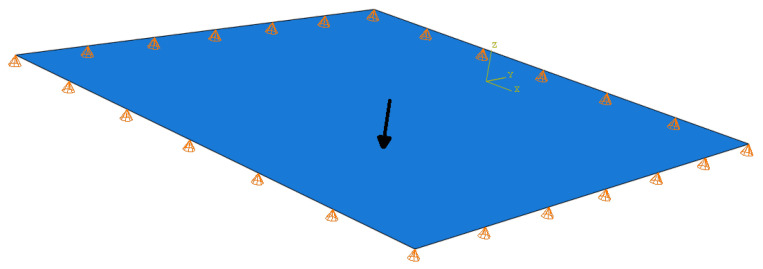
Schematic of a simply supported square plate with load and boundary conditions.

The schematic of a simply supported unit square thin plate, exposed to varying concentrated point forces of 500 N, used in this study, is shown in Figure 3. While generating snapshots for defect detection, a defect was simulated as a region with lower material property, *E*, at varying locations and sizes, compared to the surrounding area. The material parameters of a defect vary within specified ranges: $E^{i}\in \left[100,300\right]$ GPa and $\alpha^{i}\in \left[-0.25,0.25\right],\beta^{i}\in \left[-0.25,0.25\right],c^{i}\in \left[0.1,0.5\right]$. For non-defective areas, the material properties were set to E=300 Gpa, a Poisson’s ratio ν of 0.4, and a plate thickness ϵ of 1 mm.

During the search, the static displacements were computed according to the lightly intrusive ROM at each node, and they were stored for comparison with the reference displacements obtained from the finite element solver in question and including the exact defects, according to Equation (22).

### 3.1. Sensor Search

#### 3.1.1. Permutation Features Importance Method (PI)

**Figure 4:**
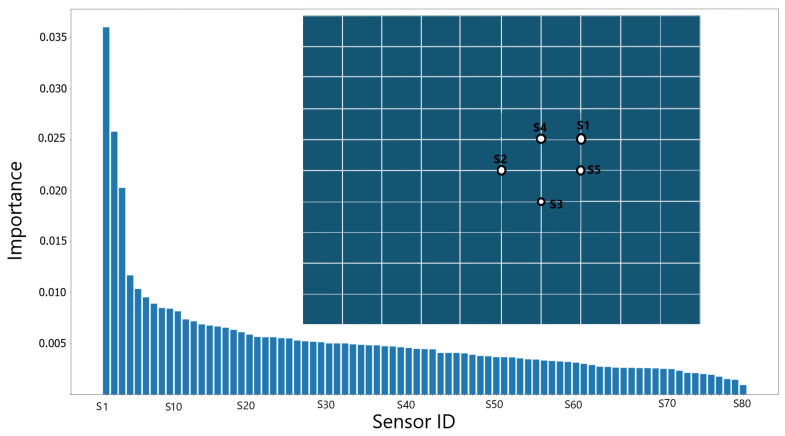
Optimal sensor locations found by permutation importance (PI).

The top five sensors identified via the PI method are depicted in Figure 4. The locations of these sensors are marked on the mesh map, with the sensor IDs indicating their ranked importance: S1 denotes the most important sensor, followed sequentially by S2, S3, and so on, in descending order of significance. Additionally, a graph illustrates the PI scores for all nodes. The PI scores reveal three leading points with a PI score exceeding 0.02. The three leading sensors are primarily situated in the middle of the mesh domain, suggesting that they are less likely to be influenced by the boundary conditions of pinning at the edges.

#### 3.1.2. Discrete Empirical Interpolation Method (DEIM)

**Figure 5:**
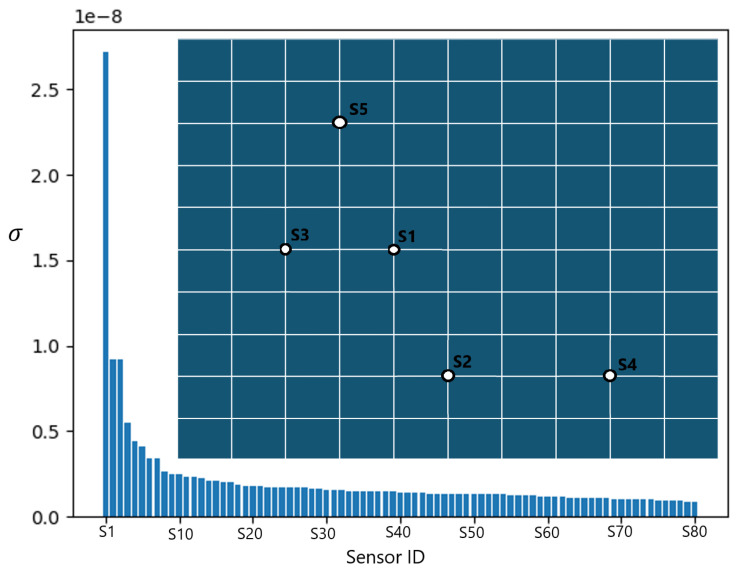
Optimal sensor locations found via the discrete empirical interpolation method (DEIM). An indirect measure of the importance of each sensor relative to the others is the singular value for each mode, denoted as σ.

The top five sensors identified using the DEIM method are depicted in Figure 5. The location of each sensor is marked on the mesh map, with the sensor IDs indicating their ranked importance. Additionally, an indirect measure of the importance of each sensor relative to the others is shown as the singular value for each POD mode, denoted as σ. The singular value of the POD mode reveals one prominent point with a significantly higher value than the other sensors. Unlike the PI findings, which primarily locate sensors in the center of the domain, the sensors found via DEIM are more dispersed, with nodes situated both in the center and elsewhere. This dispersion arises from DEIM identifying sensors related to influential modes from varying concentrated forces scenarios. Unlike PI, which focuses on sensors most sensitive to the expansion coefficients, DEIM’s approach yields sensors related to influential POD modes. Therefore, this study recommends leveraging both DEIM and PI methods, as they offer complementary insights into sensor placement.

#### 3.1.3. Hybrid

To prioritize sensors, we focused on those that ranked the highest for each method, using PI scores and the singular value of the POD modes. In this study, the highest-ranked sensor location from DEIM was selected, and the two highest-ranked sensor locations were selected from the PI method, which made for a total of three sensors for the hybrid method.

### 3.2. Defect Detection

#### 3.2.1. Sensor Confirmation

**Figure 6:**
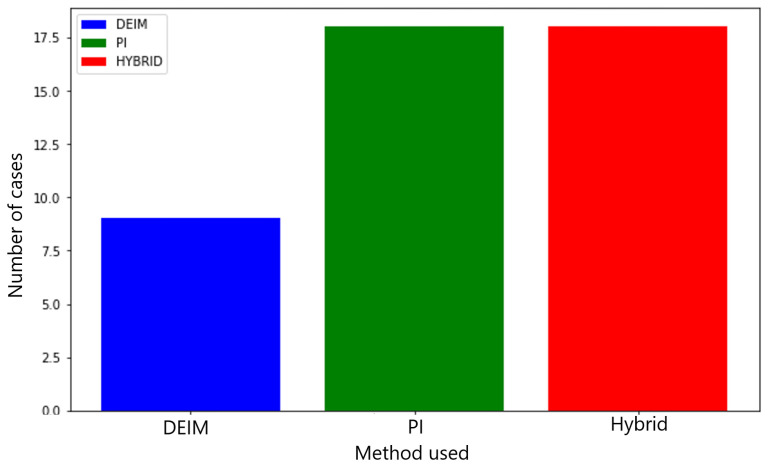
Frequency of the smallest defect detection error among the discrete empirical interpolation method (DEIM), the permutation features importance method (PI), and hybrid methods across 45 scenarios. Cases with lower error variance.

**Figure 7:**
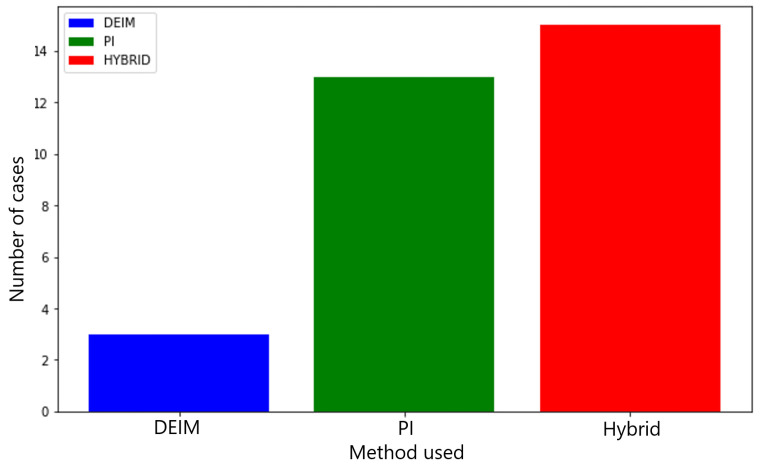
Frequency of the smallest defect detection error among the discrete empirical interpolation method (DEIM), permutation features Importance method (PI), and hybrid methods across 45 scenarios. Cases with higher error variance.

The detection error for all 45 cases was evaluated using DEIM, PI, and hybrid sensors. A total of 45 cases were designed by varying $\mu^{i}$ and the location, size, and Young’s modulus of defects: $E^{i}\in \left[100,300\right]$ GPa and $\alpha^{i}\in \left[-0.25,0.25\right],\beta^{i}\in \left[-0.25,0.25\right],c^{i}\in \left[0.1,0.5\right]$. The selected cases that were simulated in this work are listed in Table A1. The detailed test conditions are presented in the Appendix A. As shown in Figure 6, of the 45 cases, the DEIM sensor achieved the smallest error in nine cases. The PI and hybrid sensors each achieved the lowest error in 18 cases. The mean errors for all 45 cases was 5%, achieving less than 1% as the smallest error among all cases. Additionally, as shown in Figure 7, when the error variance exceeded a certain threshold, specifically 0.1% of the mean error of the three cases, the hybrid sensor identified the most cases with 15, compared to 3 cases for the DEIM sensor and 13 cases for the PI sensor.

It is important to note that using methods other than DEIM, particularly the hybrid sensors, improves defect detection accuracy. This comparison is particularly significant when the error variance among the three methods exceeds a certain threshold. In such cases, the hybrid sensors tend to produce the smallest errors in most scenarios. This suggests that, in more critical situations, where the differences in errors among the methods are more significant, the hybrid method proves to be the most effective. This highlights the effectiveness of hybrid sensors in achieving more accurate defect detection.

#### 3.2.2. Defect Detection

**Figure 8:**
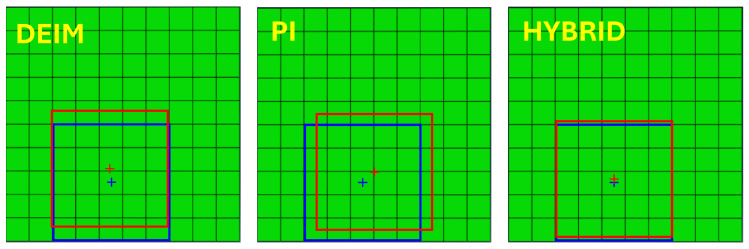
Comparison of solution (in blue) and prediction (in red) using DEIM, PI, and hybird methods.

A comparison among DEIM, PI, and hybrid sensors with one typical defect example is presented in Figure 8 and Table 1. The parameters for GA are a population size of 5, a mutation minimum value of 0.5, and a maximum value of 1. With this example, the average relative error in defect detection using hybrid sensors is the lowest. The hybrid sensor placement detects the position in the most accurate manner. Even though the detected modulus of elasticity is less accurate than what was found using the other techniques, averaging the relative errors yields the best results when using the hybrid sensor positions.

The result underscores the necessity of examining both the POD basis vectors and the expansion coefficients obtained from the SVD of the system of interest. DEIM, using the basis vectors, can identify the locations where consecutive POD modes vary the most, while PI, focusing on the global information in the solution, provides information on locations where changes in displacement significantly affect the coefficients. Both approaches contain valuable and relevant information for sensor placement.

## 4. Conclusions

This study proposes a hybrid method that utilizes the integration of both DEIM and PI methods for sensor placement, along with a defect detection method to evaluate the importance of the identified sensors. By employing DEIM and PI with the POD application of displacement snapshots, the proposed technique identifies the optimal sensor locations for defect detection. The most influential sensors from each method were then selected for a case study comparing the hybrid method against the DEIM-only or PI-only approaches. This comparison is particularly significant when the difference in detection error among the three methods exceeds a certain threshold. In these cases, the hybrid sensors consistently result in the smallest errors. This indicates that, in more critical scenarios, where the differences in errors among the three methods are more pronounced, the hybrid method performs the best. This finding underscores the advantage of the hybrid method.

The contribution of this study is twofold. First, it introduces a hybrid method for sensor displacement and confirms its effectiveness. Second, during this process, a defect detection method is proposed that is capable of computing displacement numerical solutions very quickly in a ROM setting. This combination allows for accurate sensor placement and defect detection, offering significant potential to enhance structural health monitoring practices and paving the way for more effective monitoring and maintenance strategies.

While the method was tested on simple geometries and loading conditions, its extension to complex geometries and boundary conditions does not require any further adaptation. The lightly intrusive ROM is specifically designed to reduce computational costs for industrial applications, ensuring the efficiency of the search algorithm. The current limitations stem from the ROM’s ongoing development to address nonlinear, history-dependent material behaviors like plasticity. The progress in this area is expected to enhance the hybrid method, broadening its applicability to more complex scenarios, where the normal, defect-free, structural operating conditions involve history-dependent nonlinearity.

## Figures and Tables

**Table 1:** Results for DEIM, PI, and hybrid methods. The axis origin is placed at the plate’s center.

	$x_{d}$ (m)	$y_{d}$ (m)	$l_{d}$	*E* (GPa)	Error (%)
Reference	−0.05	−0.25	0.50	272	-
DEIM	−0.055	−0.19	0.49	248	5.3
PI	0	−0.20	0.49	271	4.7
Hybrid	−0.05	−0.24	0.49	247	2.7

## Data Availability

The data presented in this study are available upon request from the corresponding author due to restrictions related to the dTHOR project.

## Appendix A

The test conditions for 45 case studies selected in this work are illustrated in Table A1.

**Table A1:** Test conditions for 45 study cases.

Case	$x_{d}$ (m)	$y_{d}$ (m)	$l_{d}$ (m)	*E* (GPa)
1	−0.25	−0.25	0.1	113
2	−0.25	−0.25	0.1	200
3	−0.25	−0.25	0.1	286
4	−0.25	−0.25	0.3	113
5	−0.25	−0.25	0.3	200
6	−0.25	−0.25	0.5	113
7	−0.25	−0.25	0.5	286
8	−0.25	−0.05	0.1	113
9	−0.25	−0.05	0.1	200
10	−0.25	−0.05	0.3	113
11	−0.25	−0.05	0.3	200
12	−0.25	0.25	0.1	113
13	−0.25	0.25	0.1	286
14	−0.25	0.25	0.5	113
15	−0.25	0.25	0.5	286
16	−0.05	−0.25	0.1	113
17	−0.05	−0.25	0.1	200
18	−0.05	−0.25	0.3	113
19	−0.05	−0.25	0.3	200
20	−0.05	−0.05	0.1	113
21	−0.05	−0.05	0.1	200
22	−0.05	−0.05	0.3	113
23	−0.05	−0.05	0.3	200
24	−0.05	−0.05	0.3	286
25	−0.05	−0.05	0.5	200
26	−0.05	−0.05	0.5	286
27	−0.05	0.25	0.3	200
28	−0.05	0.25	0.3	286
29	−0.05	0.25	0.5	200
30	−0.05	0.25	0.5	286
31	0.25	−0.25	0.1	113
32	0.25	−0.25	0.1	286
33	0.25	−0.25	0.5	113
34	0.25	−0.25	0.5	286
35	0.25	−0.05	0.3	200
36	0.25	−0.05	0.3	286
37	0.25	−0.05	0.5	200
38	0.25	−0.05	0.5	286
39	0.25	0.25	0.1	113
40	0.25	0.25	0.1	286
41	0.25	0.25	0.3	200
42	0.25	0.25	0.3	286
43	0.25	0.25	0.5	113
44	0.25	0.25	0.5	200
45	0.25	0.25	0.5	286
